# Supplementary material for: Xenopus Ssbp2 is required for embryonic pronephros morphogenesis and terminal differentiation
Source: Sci Rep. 2023 Oct 4;13:16671. doi: 10.1038/s41598-023-43662-1 (PMC10551014; doi:10.1038/s41598-023-43662-1)
Supplement: Supplementary file 1 — Supplementary Legends. [file 41598_2023_43662_MOESM1_ESM.docx]

***Xenopus* Ssbp2 is required for embryonic pronephros morphogenesis and terminal differentiation**

Ailen S. Cervino^1^, Mariano G. Collodel^1^, Ivan A. Lopez^1^, Carolina Roa^1^, Daniel Hochbaum^2^, Neil A. Hukriede^3^ and M. Cecilia Cirio^1,#^

**Supplementary figure legends**

**Supplementary Figure S1. Identification of Ldb1-Lhx1 interacting proteins by TAP in kidney-induced animal cap explants.** (**a**) Schematic of the followed procedure. 2-cell stage *Xenopus* embryos were injected with *TAP-LLCA* mRNA, cultured until blastula stage (S8) when animal caps were dissected and cultured for 6 hours in the presence of activin and retinoic acid (RA). Interacting proteins were isolated by TAP of TAP-LLCA and identified by nanoLC/MS/MS. (**b**) List of selected proteins identified in the TAP-LLCA injected sample and absent in the uninjected sample. Highlighted (grey) are proteins expressed in the pronephric kidney (Xenbase) and Ssbp2 (pink) described in this manuscript. In bold are Lhx1 and Ldb1, both part of LLCA. The number of spectral counts (SpC) is indicated for each protein. Full experimental protein list was submitted to the ProteomeXchange Consortium with the identifier PXD041761.

**Supplementary Figure S2. Validation of the *ssbp2*-MO specificity.** (**a**) Scheme of the *ssbp2-eGFP* mRNA reporter showing the *ssbp2*-MO target site. (**b-e**) 2-cell stage *Xenopus* embryos were injected into both blastomeres with 1 ng of *ssbp2-eGFP* mRNA in the presence or absence of the standard control morpholino (St-MO) or the *ssbp2*-MO (15 ng). Green fluorescence was analyzed in early gastrula stage embryos (S10.5) to allow *ssbp2-eGFP* mRNA translation. Vegetal views. (**b**) Uninjected embryos (0/40 with fluorescence). (**c**) *ssbp2-eGFP* injected embryos (39/40 with fluorescence). (**d**) *ssbp2-eGFP* + St-MO co-injected embryos (43/43 with fluorescence). (**e**) *ssbp2-eGFP* + *ssbp2*-MO co-injected embryos (2/52 with fluorescence). Note *ssbp2*-MO specifically reduced *ssbp2*-eGFP translation. Two independent experiments were performed. Representative embryos are shown.

**Supplementary Figure S3. Ssbp2 is not essential for establishment of the pronephric field.** 8-cell stage *Xenopus* embryos were injected into a single V2 blastomere as indicated. The uninjected contralateral side was used as internal control. (**a-c**) WISH for *pax8* in late neurula stage embryos (S20). Dorsal views, anterior up. (**a**) Uninjected embryo (3% affected; n = 107; N = 6). (**b**) St-MO 15 ng injected embryo (11% affected; n = 36; N = 2). (**c**) *ssbp2*-MO 15 ng injected embryo (18% affected; n = 89; N = 5). (**d-e**) WISH for *ors2* in mid-neurula stage embryos (S17). Transverse hemi sections, dorsal up. (**d**) Uninjected embryo (7% affected; n = 29; N = 2). (**e**) St-MO 15 ng injected embryo (7% affected; n = 43; N = 3). (**f**) *ssbp2*-MO 15 ng injected embryo (12% affected; n = 60; N = 3). Statistical significance was evaluated using *Chi-square* test. No significant differences were found between groups. N: number of independent experiments, n: number of embryos. Representative embryos are shown.

**Supplementary Figure S4. Ssbp2 is necessary for pronephric field morphogenesis.** 8-cell stage *Xenopus* embryos were injected into a single V2 blastomere as indicated. *Pax8* expression domain was analyzed by WISH in early tailbud stage embryos (S26) and the uninjected contralateral side was used as internal control. (**a,b**) Uninjected embryo (0% affected, n = 36, N = 3). (**c,d**) St-MO 15 ng injected embryo (14% affected, n = 29, N = 2). (**e-h**) *ssbp2*-MO 15 ng injected embryos (55% affected, n = 40, N = 3). Representative embryos are shown. N: number of independent experiments, n: number of embryos. Representative embryos are shown.

**Supplementary Figure S5. Ssbp2 depletion affects proximal and distal tubule development.** 8-cell stage *Xenopus* embryos were injected into a single V2 blastomere as indicated. The uninjected contralateral side was used as an internal control. (**a-e**) WISH for *lhx1* in 32 stage embryos. (**a**) Uninjected embryo (2% affected, n = 54, N = 3). (**b,c**) St-MO 15 ng injected embryo (5% affected, n = 56, N = 3). (**d,e**) *ssbp2*-MO 15 ng injected embryo (43% affected, n = 70, N = 3). (**f-j**) WISH for *pax8* in 32 stage embryos. (**f**) Uninjected embryo (5% affected, n = 40, N = 2). (**g,h**) St-MO 15 ng injected embryo (10% affected, n = 41, N = 2). (**i,j**) *ssbp2*-MO 15 ng injected embryo (44% affected, n = 54, N = 3). (**k-o**) WISH for *hoxb7* in stage 32 embryos. (**k**) Uninjected embryo (10% affected, n = 40, N = 2). (**l,m**) St-MO 15 ng injected embryo (31% affected, n = 35, N = 2). (**n,o**) *ssbp2*-MO 15 ng injected embryo (68% affected, n = 38, N = 2). Magnifications of the pronephric tubules enclosed by the black squares are shown in the left-bottom corner. Statistical significance was evaluated using *Chi-square* test (****p < 0.0001; ***p < 0.001). Representative embryos are shown. (**p**) Quantification of the tubule length in the most anterior *hoxb7* expression domain revealed by WISH (dotted red lines in *f-j*). The ratio between the injected and the control side is shown. Data in graph is presented as mean and standard deviation. Each point represents a single embryo. Statistical significance was evaluated using *Kruskal–Wallis* test and *Dunn’s* multiple comparisons test (**** p < 0.0001; ***p < 0.001). * represent the comparison to the uninjected group and † represents the comparison to the St-MO injected group. N: number of independent experiments, n: number of embryos.
