# Supplementary material for: Xenopus Ssbp2 is required for embryonic pronephros morphogenesis and terminal differentiation
Source: Sci Rep. 2023 Oct 4;13:16671. doi: 10.1038/s41598-023-43662-1 (PMC10551014; doi:10.1038/s41598-023-43662-1)
Supplement: Supplementary file 2 — Supplementary Figure 2. [file 41598_2023_43662_MOESM2_ESM.pdf]

Supplementary Figure S1.

a

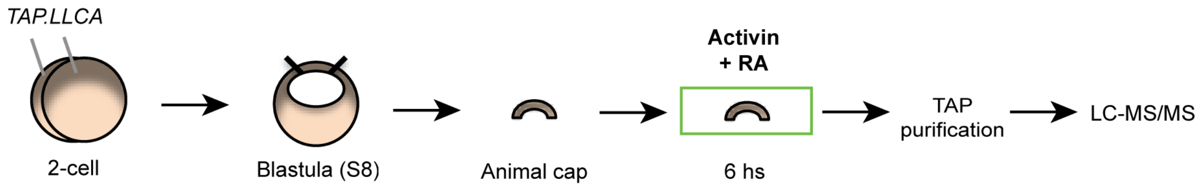

b

Identified Proteins	Accession Number	TAP.LLCA (SpC)	Uninjected (SpC)
pyruvate carboxylase, gene 2	AAI35599	71	0
propionyl CoA carboxylase, alpha polypeptide	NP_001089298	57	0
methylcrotonoyl-CoA carboxylase 1 (alpha)	NP_001086068	33	0
**LIM-domain-binding protein 1b**	BAE95405	22	0
propionyl-CoA carboxylase subunit beta L homeolog	AAH61665	21	0
tubulin alpha like 3, gene 1 L homeolog	AAH84938	17	0
methylcrotonoyl-CoA carboxylase subunit 2 S homeolog	AAI29636	17	0
Keratin, type I cytoskeletal 18-A	P08802	12	0
polyubiquitin-B precursor	AAI26016	12	0
heat shock 70kDa protein 1-like	NP_001080068	9	0
tubulin beta-4 chain	NP_001080566	9	0
receptor for activated C kinase 1 S homeolog	NP_001086465	7	0
unnamed protein product	CBF67870	7	0
NADH:ubiquinone oxidoreductase subunit A12	AAH84520	7	0
heat shock protein family D (Hsp60) member 1	AAI3584	6	0
heat shock protein 90kDa alpha, class B member 1	NP_001086624	5	0
ribosomal protein L23	NP_001085921	4	0
ATP synthase, H+ transporting, mitochondrial F1 complex, gamma polypeptide 1	NP_001080481	4	0
MGC82602 protein	NP_001085608	4	0
Vitellogenin-B2	P19011	4	0
solute carrier family 25, member 11 L homeolog	AAI23334	4	0
arginase 1	NP_001080417	3	0
single-stranded DNA binding protein 2	NP_001080347	3	0
electron transfer flavoprotein subunit alpha S homeolog	NP_001090035	3	0
NADH dehydrogenase (ubiquinone) 1 alpha subcomplex, 6	NP_001088970	3	0
NADH dehydrogenase (ubiquinone) Fe-S protein 4	NP_001087349	3	0
**LIM homeobox 1 protein**	AAI35732	3	0
40S ribosomal protein S7	P02362	3	0
mitochondrial ATP synthase beta subunit	NP_001080126	2	0
ribosomal protein S14	AAI35233	2	0
ATP synthase, H+ transporting, mitochondrial F1F0 complex, subunit e	AAI67416	2	0
tubb4 protein	AAH64270	2	0
fish-egg lectin	AAI53784	2	0
chaperonin containing TCP1, subunit 6A (zeta 1)	NP_001086080	2	0
peroxiredoxin 1 L homeolog	NP_001085178	2	0
phosphofructokinase, muscle	NP_001086921	2	0
proteasome alpha 6 subunit	NP_001086785	2	0
ribosomal protein S18	NP_001084747	2	0
ribosomal protein L30	NP_001080621	2	0
hyaluronan binding protein 4 L homeolog	NP_001083218	2	0
glyceraldehyde 3-phosphate dehydrogenase [Xenopus laevis]	AAA84422	2	0
SPT2, Suppressor of Ty, domain containing 1 [Xenopus (Silurana) tropicalis]	AAI21681	2	0
