# Supplementary figures and images for: Xenopus Ssbp2 is required for embryonic pronephros morphogenesis and terminal differentiation
Source: Sci Rep. 2023 Oct 4;13:16671. doi: 10.1038/s41598-023-43662-1 (PMC10551014; doi:10.1038/s41598-023-43662-1)

Supplementary Figure S2.

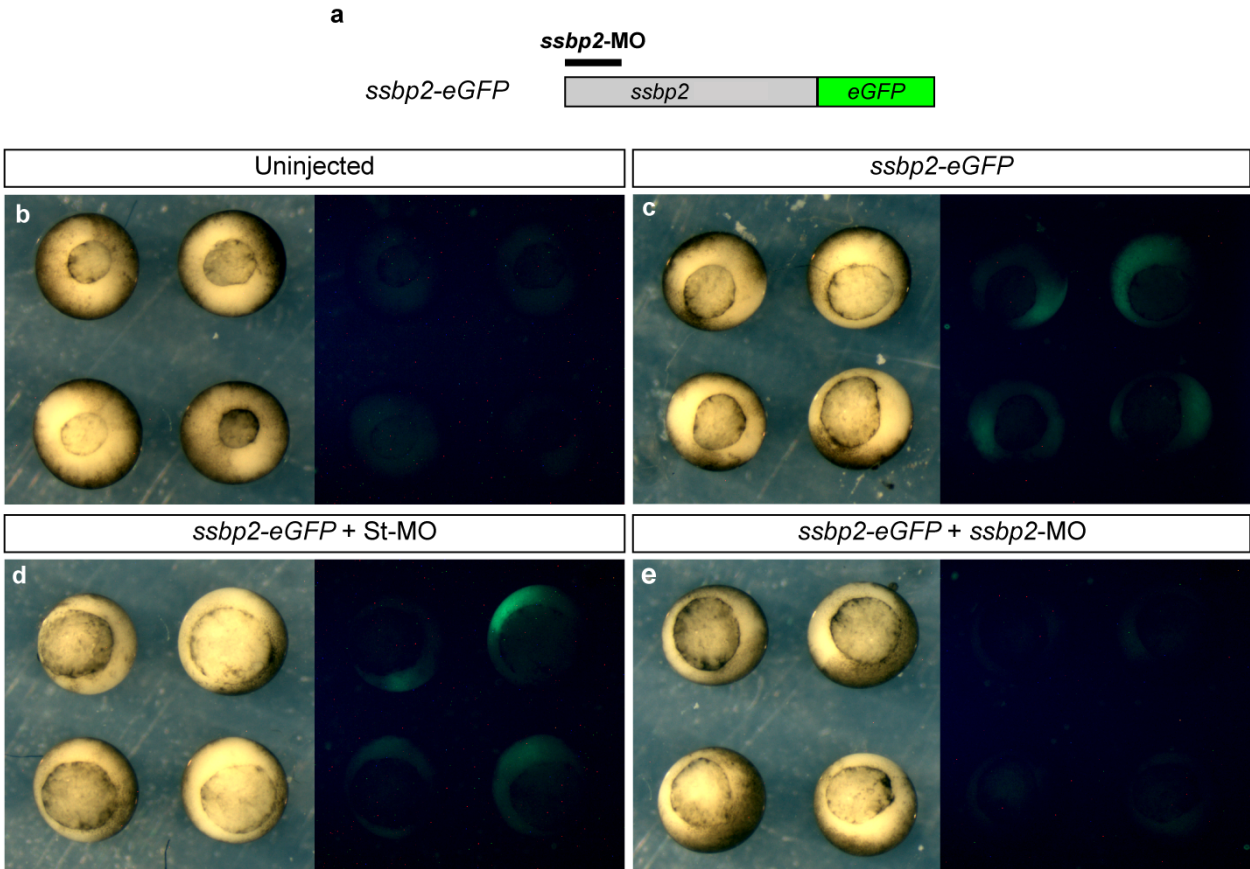

Supplement: Supplementary file 3 — Supplementary Figure 3. [file 41598_2023_43662_MOESM3_ESM.pdf]

Supplementary Figure S3.

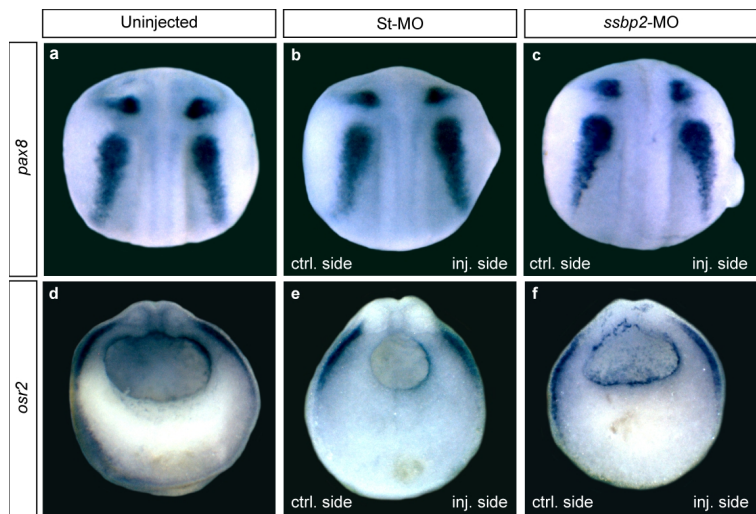

Supplement: Supplementary file 4 — Supplementary Figure 4. [file 41598_2023_43662_MOESM4_ESM.pdf]

Supplementary Figure S4.

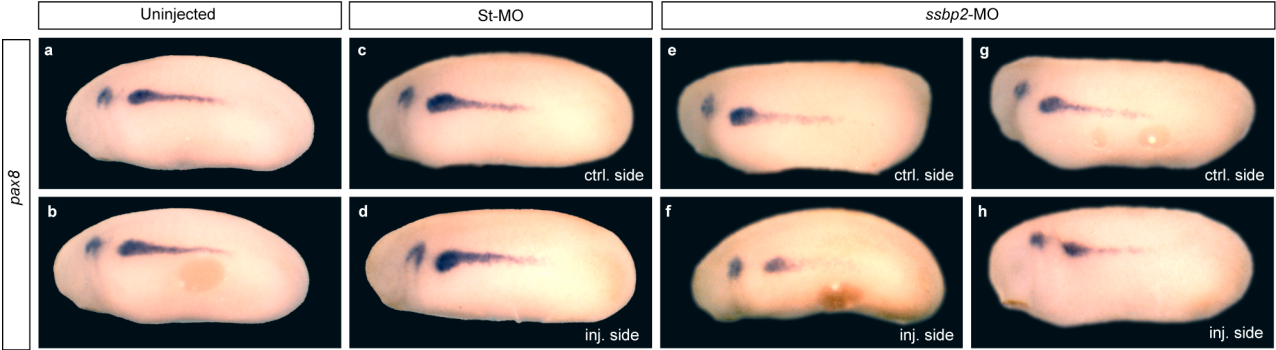

Supplement: Supplementary file 5 — Supplementary Figure 5. [file 41598_2023_43662_MOESM5_ESM.pdf]

Supplementary Figure 5.

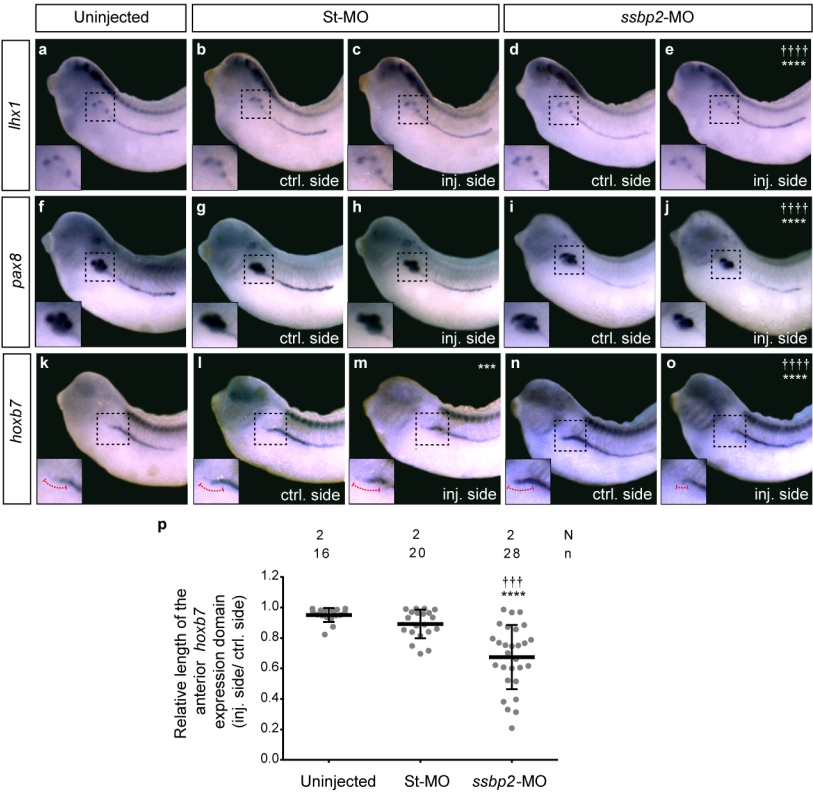

Supplement: Supplementary file 6 — Supplementary Figure 6. [file 41598_2023_43662_MOESM6_ESM.pdf]
